# The Levator Hiatus Area detected by 3D-TPUS as an indicator of rectal prolapse severity

**DOI:** 10.1007/s00384-025-05068-5

**Published:** 2026-01-09

**Authors:** Danqi Shao, Jianping Qiu, Junli Yu, Xiangwen Diao, Dan Su, Guangjian Liu

**Affiliations:** 1 Department of Ultrasonography, The Sixth Affiliated Hospital, Sun Yat-Sen University, Guangzhou, Guangdong China; 2 Biomedical Innovation Center, The Sixth Affiliated Hospital, Sun Yat-Sen University, Guangzhou, Guangdong China; 3 Department of Emergency, The First Affiliated Hospital, Sun Yat-Sen University, Guangzhou, Guangdong China; 4 Guangdong Provincial Key Laboratory of Colorectal and Pelvic Floor Diseases, The Sixth Affiliated Hospital, Sun Yat-Sen University, Guangzhou, Guangdong China; 5 Department of General Surgery (Coloproctology), The Sixth Affiliated Hospital, Sun Yat-sen University, Guangzhou, Guangdong China; 6 Department of Radiology, The Sixth Affiliated Hospital, Sun Yat-sun University, Guangzhou, Guangdong China

**Keywords:** Rectal prolapse, Dynamic three-dimensional transperineal ultrasound, Levator hiatal area enlargement, Prolapse severity, Diagnostic imaging

## Abstract

**Purpose:**

Rectal prolapse (RP) is a clinically significant condition with vaginal delivery as a major risk factor, especially in elderly females, needs precise evaluation for guiding treatment. Given the limitations of current diagnostic methods in terms of convenience, this study aims to develop an improved measure for RP.

**Methods:**

A retrospective analysis of 181 female patients undergoing both dynamic three-dimensional transperineal ultrasound (3D-TPUS) and radiographic (X-ray or MRI) defecography (X-ray and MRI) was conducted to investigate the correlation between 3D-TPUS parameters and RP severity.

**Results:**

Relative to mild RP cases, severe RP patients were older, had heavier neonatal birth weight, and less nulliparous individuals. Significant differences in severe RP cases were demonstrated by 3D-TPUS quantification, greater levator hiatal area enlargement (LHA), increased bladder neck descent (BND), and deeper rectal ampulla position (RAP) compared to mild cases. Significant predictors of severe RP identified by univariable logistic regression included age, vaginal parity, RAP, and LHA. Multivariable logistic regression analysis exhibited that age and LHA during Valsalva were the most influential indicators of severe RP. Receiver operating characteristic (ROC) curve analysis revealed that an LHA ≥ 17.5 cm^2^ is indicative for screening (sensitivity 90%, specificity 16.7%), and an LHA ≥ 32.5 cm^2^ serves as a reference threshold for surgical referral (sensitivity 26.8%, specificity 90%).

**Conclusions:**

Dynamic 3D-TPUS-measured LHA associated with with RP severity and could serve as a quantifiable marker for pelvic floor dysfunction in RP. This study introduces an adjunctive indicator for the severity of RP, improving diagnostic convenience and patient management.

**Graphical Abstract:**

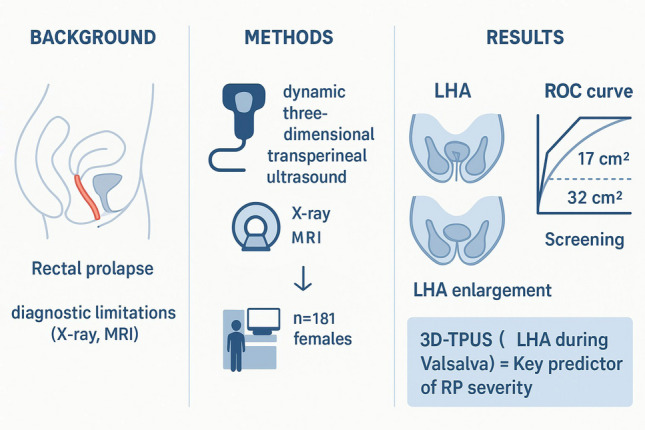

## Introduction

Rectal prolapse (RP), defined by partial or complete protrusion of the rectal wall into the intestinal lumen or through the anal canal, constitutes a clinically consequential condition linked to diverse complications [1]. Epidemiological evidence consistently identifies vaginal delivery as a primary risk factor for RP, with heightened prevalence observed in elderly female populations [2]. The underlying pathophysiology frequently involves weakening of pelvic floor muscles and intestinal support structures, commonly presenting as defecatory dysfunction [3]. Without prompt clinical intervention, progressive RP may evolve into rectal incarceration and, in advanced stages, precipitate ischemic necrosis of rectal tissues [4]. Precise evaluation and longitudinal monitoring of RP severity are therefore essential for guiding therapeutic strategies and prognostic predictions.

Current diagnostic frameworks combine symptomatic analysis, physical examination, and dynamic imaging modalities such as X-ray defecography and magnetic resonance defecography (MRD) [5]. While X-ray defecography retains its status as the reference standard for quantifying morphological changes during defecation, its clinical application is limited by radiation exposure and suboptimal soft tissue visualization [6–8]. MRD addresses these shortcomings through enhanced soft tissue contrast and multiplanar pelvic floor assessment without ionizing radiation [9], yet its routine implementation remains restricted by excessive operational costs, time-intensive protocols, and scarce availability in resource-limited regions. Given these limitations, advancing diagnostic methodologies for accurate and accessible detection of RP constitutes a critical clinical priority.

Dynamic three-dimensional transperineal ultrasound (3D-TPUS) has emerged as a pragmatic imaging alternative, gaining traction in pelvic floor evaluation due to its non-invasive nature, absence of radiation, and economic feasibility [10]. 3D-TPUS provides real-time visualization of multicompartment pelvic floor dynamics during rest and Valsalva maneuvers [10]. Utilizing high-frequency volumetric imaging, 3D-TPUS enables simultaneous anatomical measurements (e.g., levator hiatal dimensions) and functional analysis of muscular integrity, particularly within the levator ani complex and anal sphincter system [10]. 3D-TPUS procedural efficiency and cost-effectiveness support broad clinical utility, spanning initial diagnostic assessments to longitudinal monitoring of disease progression. Recent investigations further confirm the diagnostic reliability of 3D-TPUS in screening for pelvic organ prolapse [11]. To date, no studies have investigated 3D-TPUS for RP severity detection, leaving a critical knowledge gap in systematic correlations between 3D-TPUS metrics and gradations of RP severity.

To validate the utility of 3D-TPUS in quantifying the severity of RP and to establish preliminary diagnostic cutoff values, we conducted a retrospective analysis of patients who underwent both 3D-TPUS and defecography at our institution. This study systematically assessed the relationship between 3D-TPUS parameters and RP severity, aiming to inform the development of more personalized and effective management strategies for patients with RP.

## Materials and methods

### Patients

A total of 803 female patients who underwent 3D-TPUS at our center between November 2018 and June 2022 were retrospectively reviewed. Patients were eligible if they were 18 years or older and had undergone dynamic 3D-TPUS within two months of synchronous X-ray defecography or MRD. Exclusion criteria comprised: inability to cooperate during 3D-TPUS examination; incomplete defecography or MRD datasets; prior perineal/anal trauma; and postoperative recurrent rectal prolapse.

**Fig. 1:**
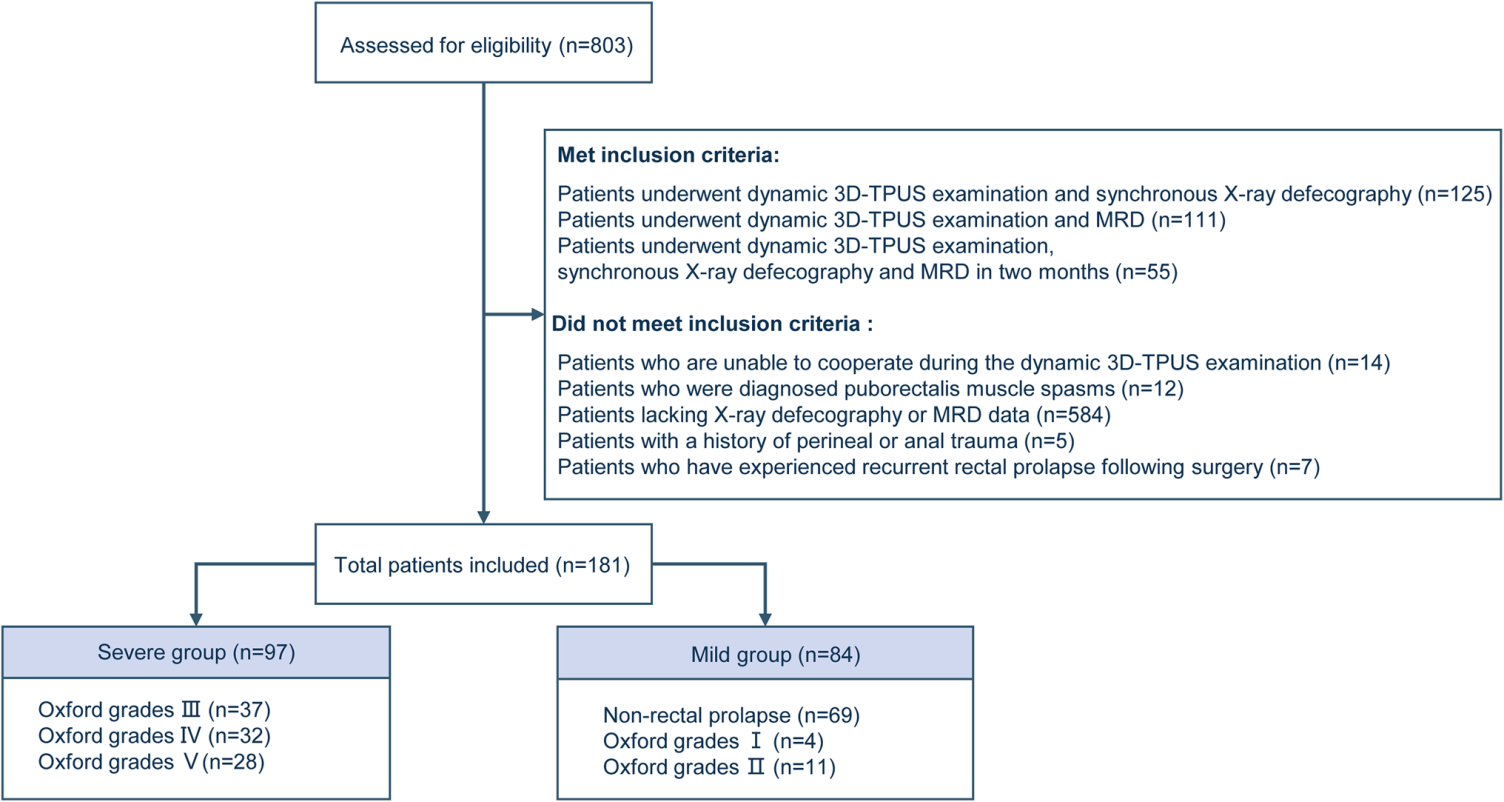
Flowchart of Patient Screening and Inclusion Criteria for rectal prolapse (RP)

The final cohort included 181 female participants categorized by defecographic findings into six groups: non-rectal prolapse and Oxford grades I-V. Consistent with guideline-defined surgical thresholds for grades III-V refractory to conservative therapy [12], a binary classification system [13] was applied based on defecography (X-ray and MRI) findings: severe group (grades III-V; n = 97) and mild group (non-prolapse/grades I-II; n = 84). This stratification reflects clinical algorithms mandating surgical evaluation for progressive rectal prolapse. The screening criteria and group allocation methodology are depicted in Fig. 1. The study protocol was reviewed and approved by the Ethics Committee of the Sixth Affiliated Hospital, Sun Yat-sen University (No. 2025ZLSYEC-277). The study received ethical approval at the analysis stage, with all data derived from previously collected, anonymized records. Written informed consent for participation was obtained from all patients prior to their inclusion in the study. All procedures were conducted in accordance with the principles of the Declaration of Helsinki, and patient data were anonymized before analysis.

### Ultrasonographic procedure

Dynamic 3D-TPUS examinations were performed using Voluson E8 and E10 ultrasound systems (GE Healthcare, Zipf, Austria) equipped with 4–8 MHz curved-array volumetric transducers operating in harmonic imaging mode. All acquisitions were conducted by certified sonographers with ≥ 5 years of specialized pelvic floor imaging experience. Two sonographers had received standardized training and were blinded to defecography and MRD findings.

Following standardized protocols, patients were positioned in lithotomy position after complete evacuation of bladder and rectal contents. The transducer was centrally aligned on the perineum along the midline sagittal plane, stabilized with minimal compression using a sterile probe cover filled with acoustic coupling gel.

Multiplanar imaging sequences were systematically acquired during three standardized functional phases: (1) resting state to establish baseline pelvic floor configuration, (2) maximal voluntary contraction with sustained pelvic floor muscle activation (≥ 3 s), and (3) Valsalva maneuver involving forced expiration against a closed glottis (≥ 6 s). To minimize variability in effort between patients during the Valsalva maneuver, all participants received standardized, professional instruction and practice prior to imaging acquisition, ensuring consistent performance across the cohort. Quantitative assessments encompassed pelvic organ descent (vertical distance from the inferior pubic symphysis margin to the bladder neck, cervix, rectal wall, measured in millimeters), levator hiatal dimensions (levator hiatal area [LHA] at rest and during Valsalva), anorectal angle (formed between the rectal longitudinal axis and anal canal central axis), and sphincter integrity (continuity evaluation of internal/external anal sphincters in axial planes) [12–16].

### Defecation proctography

Standardized bowel preparation was implemented across all participants, requiring 2-h preprocedural protocols with comprehensive patient education to ensure compliance.

X-ray defecography was performed using a Shimadzu multifunctional digital fluoroscopy system (Shimadzu Corporation, Kyoto, Japan). Following left lateral positioning, 200–300 mL of rectal contrast agent (100% w/v barium sulfate suspension; Guangzhou Guanggong Technology Development Co., Ltd., China) was administered under real-time fluoroscopic guidance. Functional evaluation proceeded using a DS-1 ergonomic defecography chair (Arcoma AB, Linköping, Sweden) with synchronized biplanar imaging (lateral and anteroposterior projections) during three phases: resting state, sustained pelvic floor contraction (≥ 5 s), and active defecation. Continuous cinefluoroscopy was recorded at 30 frames/second (70 kVp, 2.5 mAs) for offline analysis using Craestream Vue PACS (v. 12.1.5.0440).

Magnetic resonance defecography was conducted on a 1.5T superconducting MRI system (Optima MR360, GE Healthcare) with patients in supine position. Static high-resolution T2-weighted sequences were acquired in orthogonal planes. Dynamic trueFISP sequences (TR 4.5 ms, TE Min Full, temporal resolution 0.7 s/frame) captured pelvic floor kinematics during resting, contraction, and simulated defecation phases following rectal administration of 200–300 mL ultrasound gel (Guangzhou Guanggong Technology Development Co., Ltd., China) as pseudo-feces. Real-time cinematic MRI data were reconstructed using Craestream Vue PACS (v. 12.1.5.0440), with kinematic analysis restricted to studies demonstrating ≥ 80% contrast retention during defecation phase.

### Assessment of RP severity

**Fig. 2:**
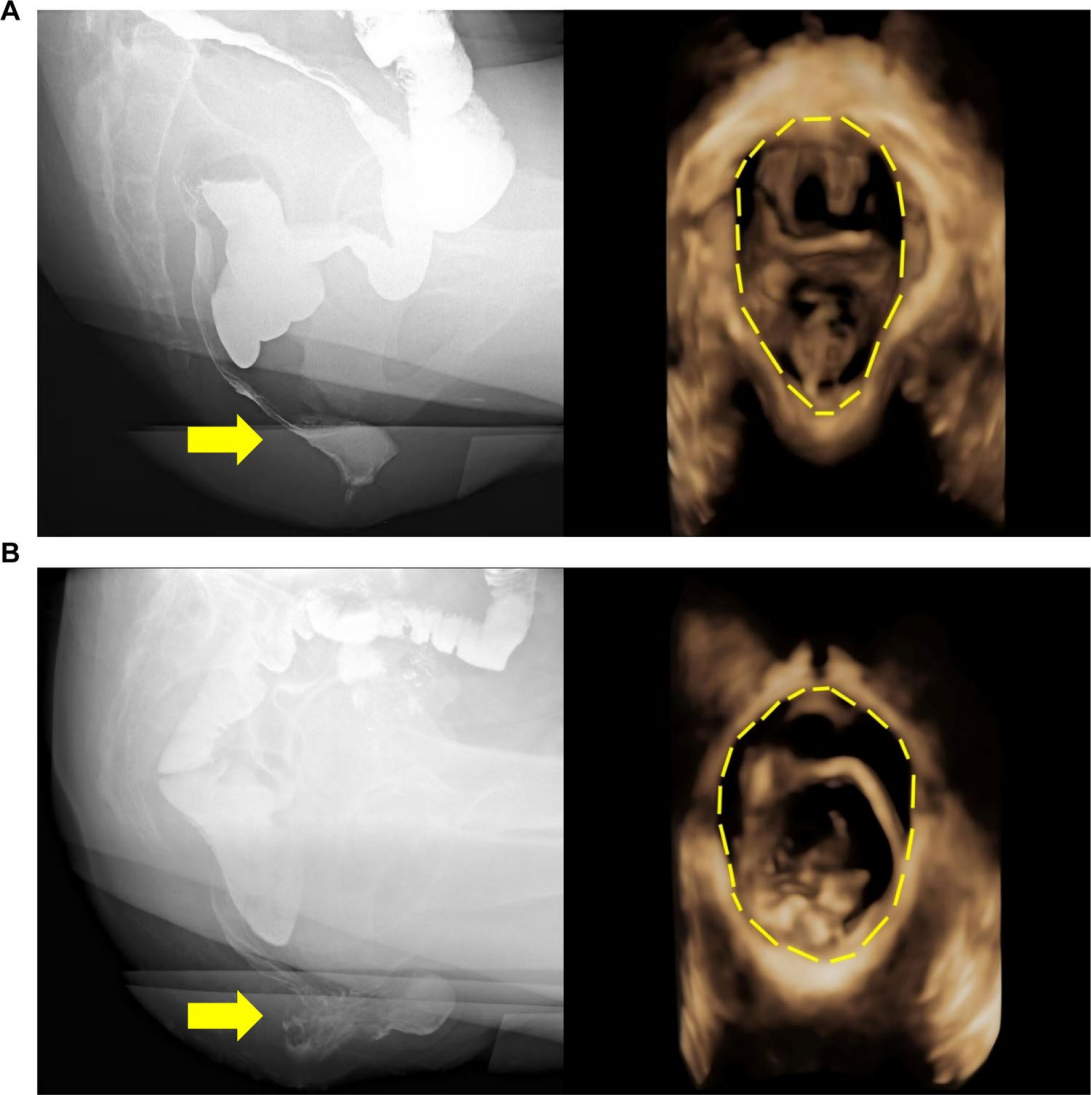
X-ray Defecography and dynamic three-dimensional transperineal ultrasound (3D-TPUS) Images of Mild and Severe Groups. X-ray defecography (recorded at 30 frames/s, 70 kV, 2.5 mA) and dynamic 3D-TPUS (penetration depth 6.1 cm, frame rate 5 Hz) of (**A**) the mild group and (**B**) the severe group

Defecographic evaluation of RP severity was conducted through blinded analysis by an experienced radiologist specializing in pelvic floor disorders, quantified maximal distal descent and applied the Oxford Radiological Grading System with the following hierarchical criteria: Grade 0 (no prolapse), Grade I (intussusception superior to proximal rectocele margin), Grade II (descent to rectocele level without anal canal engagement), Grade III (sphincter complex contact), Grade IV (intra-anal canal extension), and Grade V (complete external protrusion beyond the anal verge). Consistent with current clinical guidelines [12] that define Oxford grades III–V RP as clinically significant pathology warranting surgical management when refractory to conservative therapy or associated with quality-of-life impairment, our cohort was stratified into two severity-based categories: mild (grades 0–II; Fig. 2A) and severe (grades III–V; Fig. 2B).

### Statistical analysis

Continuous variables are expressed as mean ± standard deviation (SD) and categorical variables as counts (percentages). Normally distributed continuous variables were compared using t-test, while nonparametric data were analyzed with the Mann–Whitney U test. Univariable logistic regression identified potential predictors of RP severity based on ultrasound parameters, with variables achieving *P* < 0.05 subsequently included in a multivariable model via the enter method. Results are reported as odds ratios (ORs) with 95% confidence intervals (CIs).

The diagnostic accuracy of the RP for severe prolapse was quantified by the area under the receiver operating characteristic (ROC) curve analysis and area under the receiver operating characteristic curve (AUC). Statistical significance was defined as two-sided *P* < 0.05. All analyses were performed using SPSS (version 20.0; IBM Corp.).

## Results

### Clinical data variations between severe and mild RP groups

To elucidate the differences in clinical data among patients with varying degrees of RP, we evaluated clinical characteristics of 181 patients with RP stratified by Oxford grades into mild (n = 84) and severe (n = 97) groups (Table 1). The severe group exhibited a significantly older age profile (55.6 ± 13.6 years) compared with the mild group (48.0 ± 13.0 years; *P* < 0.001) (Table 1). Gravidity and parity distributions were close between two groups (median gravidity 3.0 pregnancies and 2.0 deliveries) (Table 1). A statistically significant disparity exists in the mode of delivery between patients with severe RP and those with mild RP (*P* = 0.01). Vaginal delivery frequency demonstrated a positive association with disease severity (severe: 89.0% [81/91], mild: 72.7% [56/77]), with Caesarean delivery rates exhibiting an inverse relationship (severe: 6.59% [6/91], mild: 10.4% [8/77]) (Table 1). A between-group disparity emerged in nulliparity, showing a significantly reduction in the severe group (4.40% [4/91]) relative to the mild group (16.9% [13/77]) (Table 1). Neonatal birth weights displayed a clinically meaningful elevation in the severe group (median 3.40 kg, range: 3.00–3.60) versus the mild group (median 3.00 kg, range: 2.84–3.50) (*P* = 0.008; Table 1). Height (*P* = 0.35) and BMI (*P* = 0.61) had consistency across severity strata (Table 1). Collectively, these results underscore pronounced differences in age, nulliparity, and birth weight between the severe and mild groups, suggesting potential clinical relevance of these indicators in assessing RP.

**Table 1:** Baseline demographic characteristics of patients with mild versus severe rectal prolapse

Variable	Total	Mild group	Severe group	*P* values
Overall	181	84	97	
Age (years)	52.1 ± 13.8	48.0 ± 13.0	55.6 ± 13.6	** < 0.001**
Gravidity, n = 175	3.0 (2.0, 4.0)	3.0 (2.0, 5.0)	3.0 (2.0, 4.0)	0.71
Parity, n = 174	2.0 (1.0, 2.25)	2.0 (1.0, 2.0)	2.0 (1.0, 3.0)	0.09
Mode of delivery	168	77	91	**0.01**
Nullipara	17 (10.1%)	13(16.9%)	4(4.40%)	
Vaginally parous	137 (81.55%)	56(72.7%)	81(89.0%)	
Cesarean	14 (8.33%)	8(10.4%)	6(6.59%)	
Birth weight (kg)	3.25 (3.00, 3.50)	3.00 (2.84, 3.50)	3.40 (3.00, 3.60)	**0.008**
Height (meter)	1.58 (1.53, 1.60)	1.58 (1.54, 1.60)	1.57 (1.53, 1.60)	0.35
BMI (kg/m^2^)	23.32 ± 3.46	23.47 ± 3.34	23.20 ± 3.56	0.61

Data are presented as n (%), median [range], or mean ± standard deviation. *P* value is a measure of the probability between mild group and severe group that an observed difference could have occurred just by random chance (*P* value was calculated with t-test or Mann-Whitney U test). A *P* value less than 0.05 is statistically significant

### Significant differences in indicators between severe and mild RP patients in 3D-TPUS examination

To further elucidate the differences between mild and severe RP groups, we assessed the parameters of 3D-TPUS for each patient and compared the values at rest and during Valsalva maneuvers. The analysis focused on the bladder neck position (BNP), cervix position, rectal ampulla position (RAP), LHA, and anorectal angle.

During the 3D-TPUS examination, distinct parameter profiles were observed between patients with severe and mild RP. BNP values at rest were similar between the two groups (*P* = 0.36; Table 2). During the Valsalva maneuver, the severe group exhibited a pronounced reduction in BNP values (− 3.0 [− 11.5, 6.50] mm) compared to the mild group (0.0 [− 7.75, 11.0] mm) (*P* = 0.042; Table 2). Cervix position remained consistent across groups at rest and during Valsalva (*P* = 0.90 and *P* = 0.98; Table 2). RAP at rest showed no significant difference (*P* = 0.10), but experienced a larger decrease in the severe group during Valsalva (*P* = 0.01) (Table 2). The LHA was significantly higher in the severe group both at rest (severe: 17.0 [14.0, 20.0] cm^2^ vs. mild: 15.0 [13.0, 17.0] cm^2^, *P* = 0.005) and during the Valsalva maneuver (severe: 27.0 [22.0, 35.0] cm^2^ vs. mild: 22.5 [20.0, 29.0] cm^2^, *P* = 0.002) (Table 2). Anorectal angle at rest or during Valsalva had no significance between severe and mild group (*P* = 0.41 and *P* = 0.50; Table 2). These findings highlight significant differences in BNP, RAP, and LHA in mild and severe groups, suggesting potential utility of perineal 3D ultrasound imaging technology in quantifying the severity of RP.

**Table 2:** Parameters of 3D-TPUS in mild versus severe rectal prolapse

Variable	Total	Mild group	Severe group	*P* values
Overall	181	84	97	
BNP (mm)				
At rest	23.0 (19.0, 27.0)	24.0 (19.3, 27.0)	23.0 (19.0, 26.5)	0.36
During Valsalva	−2.00 (−9.00, 10.0)	0.0 (−7.75, 11.0)	−3.0 (−11.5, 6.50)	**0.042**
Cervix position (mm)				
At rest	23.0 (18.3, 29.0)	22.5 (19.0, 28.8)	23.0 (18.0, 30.0)	0.90
During Valsalva	10.0 (−4.00, 17.0)	10.0 (−1.50, 15.8)	10.0 (−5.0, 18.0)	0.98
RAP (mm)				
At rest	15.0 (10.0, 19.0)	15.0 (10.0, 20.0)	14.0 (8.50, 18.0)	0.10
During Valsalva	−13.0 (−21.0, −6.0)	−11.0 (−18.0, −5.0)	−15.0 (−23.0, −8.0)	**0.01**
LHA (cm^2^)				
At rest	16.0 (14.0, 19.0)	15.0 (13.0, 17.0)	17.0 (14.0, 20.0)	**0.005**
During Valsalva	25.0 (20.0, 31.0)	22.5 (20.0, 29.0)	27.0 (22.0, 35.0)	**0.002**
Anorectal angle (º)				
At rest	118 (107, 127)	117 (105, 127)	118 (109, 127)	0.41
During Valsalva	122 (112, 136)	127 (113, 136)	122 (109, 136)	0.50

Data are presented as median [range]. *P* value is a measure of the probability that an observed difference could have occurred just by random chance (*P* value was calculated with Mann-Whitney U test). A *P* value less than 0.05 is statistically significant

### Univariate and multivariate analyses show LHA and age significantly correlated with severe RP

To determine the factors significantly associated with severe RP, we conducted univariate regression analyses. This approach enabled us to assess the individual influence of each predictor variable on RP severity. Age emerged as a significant predictor, with an OR of 1.04 (95% CI: 1.02–1.07; *P* < 0.001), indicating that for each year increase in age, the odds of severe RP increase by 4% (Table 3). Parity showed an OR of 1.18 (95% CI: 0.94–1.48; *P* = 0.16), which was not statistically significant. Among the delivery modes, vaginally parous had a notably significance OR of 4.70 (95% CI: 1.46–15.2; *P* = 0.01), suggesting a strong association with severe RP (Table 3). The LHA at rest demonstrated an OR of 1.12 (95% CI: 1.04–1.21; *P* = 0.002), and during Valsalva, the OR was 1.07 (95% CI: 1.03–1.11; *P* = 0.001), both of which were significantly associated with severe RP (Table 3). The RAP at rest showed a significant OR of 0.96 (95% CI: 0.93–0.99; *P* = 0.03), and during Valsalva, it was 0.97 (95% CI: 0.94–0.99; *P* = 0.01) (Table 3), consistent with the idea that lower RAP measurements are associated with a decreased risk of severe RP. The *P* value for connective tissue disorders was 0.731, suggesting a lack of statistical association with severe RP (Table 3). These findings underscore the importance of considering age, delivery mode, and specific anatomical measurements such as LHA and RAP in the assessment of severe RP, supported by the absence of significant differences related to cystocele, uterine prolapse, or connective tissue disorders that might otherwise influence LHA. The statistical significance of these predictors highlights their potential utility in clinical decision-making and further research.

**Table 3:** Univariable analysis of potential risk factors associated with severe RP

Variable	OR	95% CI	*P*
Age (years)	1.04	1.02, 1.07	< 0.001
Gravidity	1.02	0.88, 1.18	0.82
Parity	1.18	0.94, 1.48	0.16
Mode of delivery			
Nullipara	Ref		
Vaginally parous	4.70	1.46, 15.2	**0.01**
Cesarean	2.44	0.52, 11.4	0.26
Baby Birth weight (kg)	1.4	0.97, 2.04	0.08
Height (meter)	0.13	0.001, 26.1	0.45
BMI(Kg/m^2^)	0.98	0.90, 1.07	0.61
BNP (mm)			
At rest	0.98	0.93, 1.02	0.29
During Valsalva	0.98	0.96, 1.00	0.05
Cervix position (mm)			
At rest	1.0	0.97, 1.03	0.99
During Valsalva	1.0	0.98, 1.02	0.95
RAP (mm)			
At rest	0.96	0.93, 0.99	**0.03**
During Valsalva	0.97	0.94, 0.99	**0.01**
Connective tissue disorders	1.375	0.224, 8.45	0.731
LHA (cm^2^)			
At rest	1.12	1.04, 1.21	**0.002**
During Valsalva	1.07	1.03, 1.11	**0.001**
Anorectal angle (º)			
At rest	1.01	0.99, 1.03	0.40
During Valsalva	0.99	0.98, 1.01	0.34

*OR* odd ratio, *CI* confidence interval. *P* value is a measure of the probability that an observed difference could have occurred just by random chance. A *P* value less than 0.05 is statistically significant

To further confirm the significantly factors of severe RP and eliminate potential confounding influences, multivariable regression analysis was employed in our study. In the multivariable logistic regression model presented in Table 4, age (AOR: 1.04; 95% CI: 1.01–1.07; *P* = 0.01) and LHA during Valsalva (AOR: 1.06; 95% CI: 1.01–1.11; *P* = 0.02) were identified as significant predictors of severe RP (Table 4). These results highlight the importance of age and specific anatomical measurements in the assessment of severe RP. The analysis also supported that birth weight (AOR: 1.17; 95% CI: 0.76–1.79; *P* = 0.48), BNP during Valsalva (AOR: 1.00; 95% CI: 0.97–1.03; *P* = 0.86), and RAP during Valsalva (AOR: 0.99; 95% CI: 0.95–1.02; *P* = 0.42) were not significantly associated with severe RP (Table 4). These results indicate that, after controlling for confounding factors, only age and LHA detected by 3D-TPUS are significantly correlated with RP. Both univariate and multivariate regression analyses underscored the significance of age and LHA detected by 3D-TPUS as predictors of RP severity.

**Table 4:** Multivariable logistic regression model for severe RP risk factors model

Variable	AOR	(95% CI)	P values
Age (years)	1.04	1.01, 1.07	**0.01**
Birth weight (kg)	1.17	0.76, 1.79	0.48
BNP during Valsalva (mm)	1.00	0.97, 1.03	0.86
RAP during Valsalva (mm)	0.99	0.95,1.02	0.42
LHA during Valsalva (cm^2^)	1.06	1.01, 1.11	**0.02**

*OR* odd ratio, *CI* confidence interval. *P* value is a measure of the probability that an observed difference could have occurred just by random chance. A *P* value less than 0.05 is statistically significant

### Adjunctive diagnostic value of LHA detected by 3D-TPUS in severe RP

**Fig. 3:**
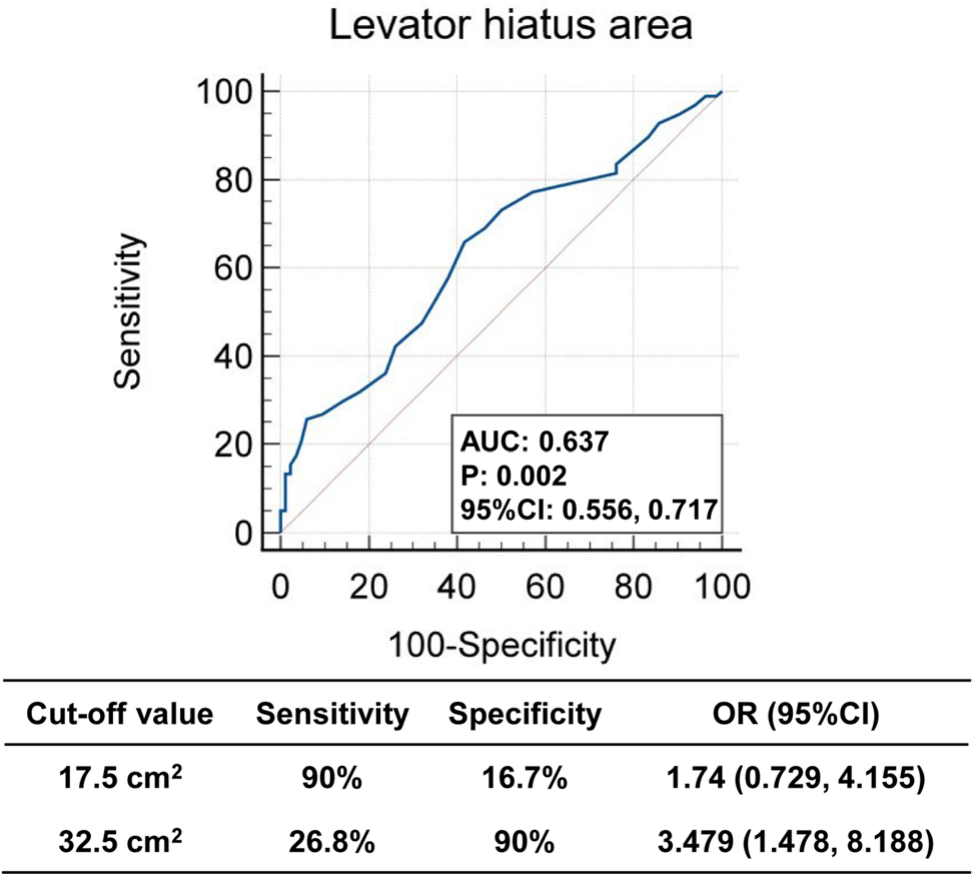
Receiver operating characteristic (ROC) curve performance of the RP and LHA Detected by 3D-TPUS. ROC curves were constructed based on the sensitivity and specificity of LHA for identifying severity of RP. The area under the receiver operating characteristic curve (AUC) was calculated based on the ROC curves and expressed as 95% CI

To explore the cutoff value of LHA for assessing RP and ensure assessment reliability, the diagnostic efficacy of the LHA detected by 3D-TPUS during the maximum Valsalva maneuver for severe RP was evaluated through ROC curve analysis. The ROC curve was employed to determine the optimal balance between sensitivity and specificity for LHA as an adjunctive marker. The AUC for LHA was 0.637 (P = 0.002), indicating a statistically significant but modest diagnostic performance for quantifying RP severity. Two specific cutoff values for LHA were identified (Fig. 3). At 17.5 cm^2^, LHA demonstrated a high sensitivity of 90% but low specificity of 16.7%; at 32.5 cm^2^, sensitivity decreased to 26.8%, with specificity rising to 90%. These findings suggest that LHA measurements during the Valsalva maneuver provide valuable information for assessing severe RP, despite the moderate overall diagnostic accuracy (Fig. 3). Based on these findings, LHA thresholds of ≥ 17.5 cm^2^ for screening and ≥ 32.5 cm^2^ for surgical referral were established (Fig. 3). These findings provide quantitative evidence in the reference value of LHA, highlighting its potential utility in clinical decision-making for evaluating severe RP, suggest that measuring LHA detected by 3D-TPUS during the Valsalva maneuver holds promise as a valuable adjunctive indicator for severe RP.

## Discussion

RP, a benign disorder defined by full-thickness or partial extrusion of the rectal wall beyond the anal canal, demonstrates a markedly higher incidence in females compared to males [17]. The global demographic shift toward an aging population has contributed to rising prevalence rates and clinical severity of this condition [18]. Affected individuals commonly present with debilitating symptomatology encompassing anal discomfort, hemorrhage, constipation, and fecal incontinence [19]. While not directly life-threatening, RP imposes substantial detrimental impacts on health-related quality of life, underscoring the imperative for precise diagnostic evaluation [20]. This study first establishes the clinical utility of 3D-TPUS in quantifying RP severity. Our data demonstrates that 3D-TPUS enables objective measurement of LHA, serving as an adjunctive indicator for severe RP. This advancement carries substantial clinical relevance by introducing a non-invasive, radiation-free diagnostic modality with high reproducibility. The implementation of this technique is projected to enhance therapeutic decision-making, optimize patient outcomes through early intervention, and ultimately mitigate disease-associated morbidity.

A pivotal discovery in this study was the substantial correlation between LHA, especially during maximum Valsalva, and the extent of RP. The LHA acts as a proxy for the integrity of the pelvic floor support system, encompassing the levator ani muscles, connective tissues, and innervation network [21]. Deficits in the structure or function of these components—be it due to trauma, aging, or neuropathic degeneration—disrupt pelvic floor biomechanics, resulting in hiatal widening [22]. Research indicates that levator hiatus enlargement constitutes a significant etiological factor in pelvic organ prolapse among female populations [23]. Furthermore, the correlation between LHA and RP builds on previous observations regarding pelvic floor morphometry-prolapse relationships. For instance, Dietz et al. [22] identified Valsalva-phase LHA as a quantifiable predictor of both rectal intussusception and advanced-stage POP (Pelvic Organ Prolapse Quantification [POP-Q] ≥ 2), demonstrating moderate discriminative capacity (AUC 0.76; sensitivity 52%, specificity 83% at 25 cm^2^ cutoffs). Our investigation specifically targets posterior compartment pathology, no prior studies have specifically examined the relationship between LHA and RP severity. In the present study, through systematic validation, adjunctive indicator for RP assessment have been established: LHA (levator hiatus area, quantified by rectal ampullary cross-sectional measurement) ≥ 17.5 cm^2^ for screening detection and ≥ 32.5 cm^2^ as a reference for surgical referral criteria. This evidence-based standardization enhances the clinical utility of 3D-TPUS in pelvic floor diagnostics, supporting data-driven therapeutic decision-making. However, the LHA cut-off of ≥ 17.5 cm^2^, although highly sensitive, exhibits limited specificity and may be applied as a screening parameter to exclude severe RP, with positive results requiring confirmation by additional assessment. The LHA threshold of ≥ 32.5 cm^2^ demonstrates higher specificity but remains insufficiently accurate to serve as a standalone criterion for surgical referral. ROC analysis revealed an AUC of 0.637 for LHA during the Valsalva maneuver, likely due to the complex and multifactorial etiology of rectal prolapse. As LHA primarily reflects pelvic floor laxity rather than prolapse-specific structural changes, its specificity for assessing RP severity is inherently limited.

There are several limitations in this study. First, the requirement for patients to undergo both 3D-TPUS and defecography was necessary to ensure a rigorous comparison between ultrasound and the reference standard. However, this inclusion criterion may have introduced selection and verification biases by excluding patients who were clinically diagnosed without defecography or did not require comprehensive imaging. Consequently, the study population may not fully represent the broader RP patient population, potentially limiting the generalizability of the findings. Second, 3D-TPUS is an operator-dependent technique, and imaging performed in the lithotomy position, although consistent with AIUM and IUGA guidelines, may not fully replicate physiological pelvic floor dynamics, potentially underestimating the maximal descent in this gravity-dependent condition. Third, although all examinations were conducted by experienced sonographers following standardized protocols in accordance with AIUM and IUGA pelvic floor ultrasound guidelines, intra- and interobserver variability was not formally assessed. The absence of a structured reproducibility analysis limits the ability to determine measurement consistency across different operators or repeated assessments, and future prospective studies incorporating standardized reliability testing are warranted to address this gap. Finally, although our results suggest that LHA may serve as an indicator of rectal prolapse severity, the observed associations are correlational and should be interpreted with caution. Future multicenter prospective studies with broader inclusion criteria, physiological examination platforms, and formal reproducibility testing are needed to validate and extend these findings.

In conclusion, our study demonstrates that LHA measurements via dynamic 3D-TPUS correlate with RP severity, suggesting its potential as an adjunctive indicator of pelvic floor dysfunction for clinical decisions. These findings offer novel perspectives for evaluating and monitoring advanced RP, especially in patients requiring therapeutic interventions. However, these conclusions remain preliminary due to the single-center retrospective design and inherent technical variations in 3D-TPUS measurements. Thus, multicenter prospective studies with standardized protocols are required to validate the clinical utility of LHA as an adjunctive indicator for assessing RP severity.

## Data Availability

The data that support the findings of this study are available from the corresponding author upon reasonable request.
